# Advancing arbovirus diagnosis in Brazil: strengthening diagnostic strategies and public health data collection

**DOI:** 10.1016/j.bjid.2024.103766

**Published:** 2024-05-25

**Authors:** Brena F. Sena, Bobby Brooke Herrera, Danyelly Bruneska Gondim Martins, José Luiz Lima Filho

**Affiliations:** a Keizo Asami Institute, Universidade Federal de Pernambuco, Recife, PE, Brazil; b Rutgers Robert Wood Johnson Medical School, Department of Medicine, Division of Allergy, Immunology, and Infectious Diseases, and Child Health Institute of New Jersey, Rutgers University, NJ, USA; c Rutgers University, Rutgers Global Health Institute, NJ, USA; d Department of Biochemistry, Universidade Federal de Pernambuco, Recife, PE, Brazil

**Keywords:** Arboviruses, Co-infections, Diagnostics, Public health data, Major tropical diseases

## Abstract

**Background:**

The last five decades have seen a surge in viral outbreaks, particularly in tropical and subtropical regions like Brazil, where endemic arboviruses such as Dengue (DENV), Zika (ZIKV), and Chikungunya (CHIKV) pose significant threats. However, current diagnostic strategies exhibit limitations, leading to gaps in infection screening, arbovirus differential diagnoses, DENV serotyping, and life-long infection tracking. This deficiency impedes critical information availability regarding an individual's current infection and past infection history, disease risk assessment, vaccination needs, and policy formulation. Additionally, the availability of point-of-care diagnostics and knowledge regarding immune profiles at the time of infection are crucial considerations.

**Objectives:**

This review underscores the urgent need to strengthen diagnostic methods for arboviruses in Brazil and emphasizes the importance of data collection to inform public health policies for improved diagnostics, surveillance, and policy formulation.

**Methods:**

We evaluated the diagnostic landscape for arboviral infections in Brazil, focusing on tailored, validated methods. We assessed diagnostic methods available for sensitivity and specificity metrics in the context of Brazil.

**Results:**

Our review identifies high-sensitivity, high-specificity diagnostic methods for arboviruses and co-infections. Grifols transcription-mediated amplification assays are recommended for DENV, CHIKV, and ZIKV screening, while IgG/IgM ELISA assays outperform Rapid Diagnostic Tests (RDTs). The Triplex real-time RT-PCR assay is recommended for molecular screening due to its sensitivity and specificity.

**Conclusion:**

Enhanced diagnostic methods, on-going screening, and tracking are urgently needed in Brazil to capture the complex landscape of arboviral infections in the country. Recommendations include nationwide arbovirus differential diagnosis for DENV, ZIKV, and CHIKV, along with increased DENV serotyping, and lifelong infection tracking to combat enduring viral threats and reduce severe presentations.

## Introduction

The past five decades have witnessed a staggering rise in viral outbreaks, particularly in tropical regions like Brazil. Among these outbreaks, Dengue Virus (DENV) has emerged as a persistent threat to the Brazilian population. The scale of the issue is illustrated by recent figures: Brazil reported an alarming 5 million cases of DENV infections in just the first five months of 2024, a significant increase from the 1.6 million cases reported throughout 2023.^1^ Several factors contribute to this concerning trend, including rising temperatures, the broader impact of climate change, rapid population growth, and increased global connectivity through travel and trade.^2^

Simultaneously, Brazil continues to grapple with outbreaks of Zika Virus (ZIKV) and Chikungunya Virus (CHIKV), with 3,600 cases of ZIKV and 135,000 cases of CHIKV reported in the early months of 2024.^1^^,^^3^ Complicating matters further, all four DENV serotypes (DENV-1, DENV-2, DENV-3, and DENV-4) co-circulate in the country, leading to dynamic infection patterns that impact immune response and disease severity.^4^^,^^5^ Recent studies have also highlighted the prevalence of co-infections among these viruses in Brazil.^6^, ^7^, ^8^ However, existing diagnostics and data collection efforts do not fully capture this complex interplay. The high co-infection rate among DENV, ZIKV, and CHIKV highlight the need for a more refined differential diagnostic approach that considers genetic similarities and can detect the viruses at high sensitivity and specificity.

Understanding the dynamics of DENV infection and seropositivity is crucial, particularly given the phenomenon of “original antigenic sin” and “antibody-dependent enhancement”, which can lead to severe outcomes such as Dengue Hemorrhagic Fever/Dengue Shock Syndrome (DHF/DSS) following a subsequent infection with a different, but genetically related flavivirus.^9^, ^10^, ^11^, ^12^, ^13^, ^14^ Moreover, ZIKV gained global attention starting in 2015 due to significant outbreaks in Latin America, and its association with microcephaly and neurological damage in newborns, further highlighting the urgency of addressing these viral threats.^3^

Despite these challenges, comprehensive data on the shifting patterns among DENV, ZIKV, and CHIKV remain limited. This knowledge gap extends to both individual and population-level understanding, as well as differential diagnostic strategies. Currently, Brazil relies on serologic assays detecting IgM antibodies and PCR-based methodologies to diagnose acute arboviral infections. However, gaps persist in identifying previous infections, specific serotypes, and the availability of point-of-care methods, hindering comprehensive diagnostics. Furthermore, information regarding diagnostic test used and performance, long-term infection tracking, and immune profiles of affected individuals is lacking.

This review aims to bridge these knowledge gaps by providing a comprehensive overview of arboviral diagnostics in Brazil, focusing on validated diagnostic tools and their implications for public policy. By addressing these gaps in understanding and emphasizing the importance of enhanced diagnostic strategies and data collection, we aim to strengthen Brazil's public health response to these multifaceted viral threats.

## Methods

This review focused on evaluating key diagnostic tools for the detection and differential diagnosis of arboviral infections caused by DENV, CHIKV, and ZIKV, with a spotlight on those validated and relevant to Brazil (Table 1). Our assessment encompassed a spectrum of methods, ranging from singleplex assays for DENV detection to advanced multiplex tests capable of identifying DENV, ZIKV, and CHIKV simultaneously. We systematically evaluated the performance metrics of these diagnostic strategies, including sensitivity, specificity, and sample size considerations, to glean insights into the efficacy.

**Table 1:** Main studies in diagnostic methods and assays for arboviral infections in Brazil.

Study Reviewed	Type of Study	Study Design	Method of Analysis	Population studied & number of samples	Sensitivity / Specificity	Key Findings and Conclusions
Stone et al., 2023^18^	Performance Evaluation	Nucleic Acid Tests	Nucleic acid amplification tests for DENV, CHIKV, and ZIKV	1017 plasma samples from individuals with DENV and suspected ZIKV infection	Sensitivity: 95.39%, Specificity: 100%.	Grifols assays (single plex and multiplex) showed high sensitivity. Grifols transcription-mediated amplification (TMA) assays recommended for screening in arbovirus outbreak regions.
Pereira et al., 2023^23^	Performance Evaluation	ELISA	ELISA for CHIKV IgM and IgG	660 samples from individuals suspected of CHIKV infection	IgM Sensitivity: >88%; IgG Sensitivity: 100%; Specificity: 100%.	CHIKV IgG/IgM ELISA exhibited high sensitivity and specificity. Outperformed RDT in accuracy.
Tsai et al., 2023^24^	Performance Evaluation	ELISA, IgG-capture ELISA	DENV IgG ELISA, and IgG-capture ELISA	232 serum or plasma samples, including late-convalesce ZIKV samples from Brazil.	IgG ELISA Sensitivity: 94.9%; IgG ELISA Specificity: 100%; IgG-capture ELISA Sensitivity: 18.6%; IgG-capture ELISA Specificity: 100%.	Compared DENV IgG and IgG-capture ELISAs using serum/plasma panels with flavivirus infections. IgG ELISA showed higher sensitivity overall, suggesting its suitability in seroprevalence studies and pre-vaccination screening for dengue vaccines.
DiazGranados et al., 2021 ^25^	Performance Evaluation	ELISA, Rapid Diagnostic Test (RDT)	DENV IgG ELISA, and RDT	3833 samples, 2486 IgG-RDT positive	Sensitivity: 91.1%, Specificity: 92.8%,	IgG RDT demonstrated exceptional sensitivity and specificity. Suitable for screening DENV history and pre-vaccination seropositivity.
Ribeiro et al., 2021^15^	Performance Evaluation	RT-PCR	ZDC Molecular Assay for DENV, CHIKV, ZIKV	269 plasma samples	Sensitivity: 100%, Specificity: 100%.	ZDC molecular RT-PCR assay exhibited high sensitivity and specificity for detecting DENV, CHIKV, and ZIKV.
Morales et al., 2021^26^	Performance Evaluation	ELISA	IgM, IgAM, and IgG ELISAs for ZIKV antibodies	543 samples from patients in Brazil	IgAM Sensitivity: 93.5%, IgAM Specificity: 85%, IgM Sensitivity: 30.3%, IgM Specificity: 93%, IgG Sensitivity: 72%, IgG Specificity: 100%.	IgAM ELISA showed strong performance during co-circulation of ZIKV, DENV, and CHIKV; where IgG displayed strong performance compared to IgM in a population faced with co-infection.
Pereira et al., 2021^27^	Performance Evaluation	ELISA	NS1-based DENV IgG ELISA	76 serum samples	Sensitivity: 82%, Specificity: 93%.	Dengue IgG ELISA test showed promising accuracy with a good sensitivity (82%) and specificity (93%), making it effective for spotting DENV-specific antibodies; no cross-reactivity with ZIKV NS1 was found.
Kikuti et al., 2019^28^	Performance Evaluation	RDT	DENV NS1, IgM, IgG ELISA vs. RDT	500 serum samples	Sensitivity: 46.8%, Specificity: 96%.	Dengue Duo RDT showed high specificity but lower sensitivity for NS1 or IgM. Cross-reactions were not assessed.
Colombo et al., 2019^16^	Performance Evaluation	RT-PCR	Trioplex real-time RT-PCR	1656 serum samples	Sensitivity: 95.39%, Specificity: 100%.	Trioplex RT-PCR detects DENV, CHIKV, and ZIKV with high sensitivity and specificity. Recommended for molecular screening in Brazil.

For arbovirus detection, our study evaluated a diverse set of diagnostic methods, including NS1 protein detection in antigen-based tests, ELISA-based assays, and RT-PCR based assays. To ensure a comprehensive examination of the literature, we conducted a rigorous search across multiple databases, including PubMed, Web of Science, Google Scholar, SciELO, LILACS and EMBASE, and Scopus. Our search strategy encompassed relevant articles published up to present employing a combination of keywords such as “diagnostics”, “DENV”, “CHIKV”, “ZIKV”, “Brazil”, “arboviruses”, “performance”. We included studies which assessed test sensitivity and specificity, and in the context of Brazil. We excluded those that did not assess assay performance.

## Results

Our review of the literature revealed a diverse array of diagnostic methodologies available for DENV, ZIKV, and CHIKV, including antigen, serological, and molecular-based assays (Table 1). These encompassed detection methods ranging from the NS1 protein in flavivirus antigen-based tests to IgG/IgM antibody-based or ELISA-based tests, as well as the detection of viral RNA through PCR-based methods. Despite the availability of various diagnostic approaches, our findings suggest a scarcity of rapid point-of-care diagnostic options within the Brazilian public health system.

Upon reviewing the diagnostic methods, particularly those validated in Brazil, we identified several high-performing options that should be incorporated into the country's diagnostic strategy (Table 1). Most of these methods exhibited robust sensitivity and specificity for detecting DENV, ZIKV and CHIKV, with RT-PCR emerging as the primary method for distinguishing between these viruses.

Notably, studies by Ribeiro et al. (2021) ^15^ and Colombo et al. (2019) ^16^ provided significant evidence supporting the efficacy of certain RT-PCR assays, especially during outbreaks. The ZDC molecular assay ^15^ showed promise in detecting positive samples for DENV, ZIKV, and CHIKV with high sensitivity (100%) and specificity (100%) in clinical performance. However, the clinical samples evaluation included a sample size of only 228 plasma samples, which could benefit from a more robust cohort for assessing performance.

In an evaluation of the CDC Trioplex RT-PCR assay for detecting ZIKV, DENV, and CHIKV RNA, researchers found it to demonstrate high performance with 95% sensitivity and 100% specificity in a robust set of clinical samples, analyzing 1,656 serum samples.^16^ These samples were from symptomatic individuals with acute febrile disease for 5 days or less.

Other studies, such as those by Pereira et al. (2023) and Morales et al. (2021), highlighted the success of ELISA-based tests for CHIKV and ZIKV detection incorporating both IgG and IgM antibodies. DiazGranados et al. (2021) evaluated a commercially available immunoassay for DENV IgG detection, with the EUROIMMUN Anti-Dengue Virus NS1 Type 1‒4 ELISA (IgG) showing the highest performance with 88.2% sensitivity and 98.8% specificity. This assay is one of the two IgG tests currently used by the US CDC in a two-test algorithm for determining previous DENV history and vaccine eligibility based on the presence of IgG antibodies.^17^

Finally, Stone et al. (2023) evaluated clinical specimens collected from individuals with dengue-like syndrome or suspected ZIKV infection in Brazil. They found that the Grifols transcription-mediated amplification TC-TMA assay demonstrated increased sensitivity for ZIKV, DENV, and CHIKV compared to RT-qPCR, with a robust clinical sample size of 1,017 individual samples evaluated. Additionally, the LAMP technologies by Gomes et al. (2020) offers a unique advantage for viral detection without requiring a thermal cycler, an advancement to RT-PCR technologies.

## Discussion

The pressing need to strengthen diagnostic methods in Brazil cannot be emphasized enough. Notably, Brazil's current diagnostic approach lacks the detection of DENV IgG antibodies, crucial for identifying past infections. By incorporating techniques such as arboviral serology including IgG screening, comprehensive DENV serotyping, and differential diagnostics for DENV, CHIKV, and ZIKV nationwide, we can gain a nuanced understanding of infection dynamics, and potential severity. Presently, heavy reliance on clinical presentations and IgM-based antibodies for arboviral screening falls short of capturing the true breadth of infections in Brazil. The transient nature of IgM antibodies, coupled with asymptomatic cases and undetectable fever symptoms at the point of care, renders them inadequate indicators of past or current infections.

Understanding the DENV seropositivity is pivotal in shaping effective public health strategies, especially in regions where vaccination programs depend on prior infection assessments to mitigate the risks of severe diseases upon subsequent infections.

The multiplex transcription-mediated amplification Grifols assay has demonstrated promising sensitivity in identifying cases of DENV, CHIKV, and ZIKV,^18^ outperforming conventional methods such as RT-qPCR. Its strength lies in its evaluation within a robust clinical sample size during ZIKV and DENV outbreaks, and its capability to differentiate between the three arboviruses.

While some RT-PCR assays developed in Brazil, like the ZDC molecular assay ^15^ have garnered approval from the Brazilian regulatory agency (ANVISA) for commercial availability, their clinical performance evaluation could benefit from a larger sample size encompassing larger numbers of DENV, CHIKV, and ZIKV cases.

Furthermore, the availability of high-performing IgG-based methodologies holds significant value in Brazil, given the expected exposure of a large portion of the population to arboviral infections. IgG ELISA assays for DENV 1‒4, CHIKV, and ZIKV detection, including the DENV 1‒4 IgG ELISA assay implemented by the US CDC, offer crucial insights into previous infection history, particularly pertinent ahead of vaccination efforts.

Recent research has unveiled that prior DENV infection may confer a degree of immunity against subsequent ZIKV infections,^19^ with timing between infections playing a pivotal role.^4^^,^^20^^,^^21^ These findings have profound implications for vaccination strategies, particularly in multi-viral prevalent regions like Brazil, where screening for previous infections with IgG detection could optimize vaccination efforts.

Moreover, the emergence of LAMP technologies, such as that proposed by Gomes et al.,^22^ offers a promising alternative to PCR for point-of-care molecular diagnostics. Its ability to amplify DNA and RNA at stable, low temperatures, promises faster results, eliminating the need for the thermal cyclers pivotal for PCR. Identifying individuals at heightened risks for progressing to severe conditions equips healthcare professionals with the necessary tools for better screening, tracking, and disease management.

## Conclusion

In conclusion, enhancing the diagnostic methods for arboviral infections, particularly DENV, CHIKV, and ZIKV in Brazil, is paramount. Given the potential for IgM antibodies to provide a limited snapshot due to their transient nature, integrating IgG-antibody detection to identify past infections, DENV serotyping, and differential diagnosis strategies offers a more complete insight into infection rates and potential severity. Understanding DENV past infections, and seropositivity is critical, especially in areas where vaccination strategies require prior infection history to prevent severe outcomes from subsequent infections. The silent nature of many arboviral infections emphasizes the need to move beyond symptoms-based diagnoses, towards the implementation of high-performing diagnostic methods.

Furthermore, Brazil's pressing challenge lies in addressing the dynamic patterns of infection rates. The widespread nature of arboviral infections necessitates methodical diagnosis and consistent monitoring. By bridging diagnostic gaps, Brazil can take robust measures towards managing and mitigating the impacts of arboviral infections in the country. Efforts towards public health, education, and resource allocation are essential to raise awareness about these viral agents’ risks and consequences, ensuring informed public policy initiatives and effective disease management that can affect global health.

## Conflicts of interest

The authors declare that the research was conducted in the absence of any commercial or financial relationships that could be construed as a potential conflict of interest.
